# Nanomaterials and immune checkpoint inhibitors in cancer immunotherapy: the synergistic innovation prospects

**DOI:** 10.3389/fimmu.2025.1582774

**Published:** 2025-06-04

**Authors:** Jing Chen, Xue Cheng

**Affiliations:** Medical Oncology Department of Thoracic Cancer, Cancer Hospital of Dalian University of Technology, Cancer Hospital of China Medical University, Liaoning Cancer Hospital & Institute, Shenyang, Liaoning, China

**Keywords:** nanomaterials (A), cancer, immune checkpoint inhibitors, immunotherapy, prospects

## Abstract

Tumor cells can utilize the immune checkpoint pathway to inhibit T cell activation and evade the attack of tumor specific T cells. While immune checkpoint inhibitors (ICIs) competitively bind to checkpoint molecules to block checkpoint mediated suppression of the immune system. Immune checkpoint inhibitors have emerged as a milestone in cancer immunotherapy, demonstrating significant efficacy in various cancers. However, their clinical application still faces challenges such as low response rates (<30% in solid tumors), significant side effects, and suppression by the tumor microenvironment. Nanomaterials offer new solutions to optimize the therapeutic effects of ICIs. This article explores the potential of combining nanomaterials with ICIs in cancer treatment.

Firstly, nanomaterials can reduce the side effects of ICIs. Nanomaterials can be used to deliver ICIs, thereby improving the bioavailability of the drugs. The systemic distribution of ICIs (such as anti-PD-1/PD-L1 antibodies) may lead to immune attacks on normal tissues, causing severe side effects. Through precise delivery, nanomaterials like hydrogels can reduce the systemic exposure of ICIs, thereby lowering the incidence of immune-related adverse reactions (4). For example, Wilson et al. developed a nanoparticle delivery system using biodegradable poly(β-amino ester) nanoparticles to co-deliver a STING agonist (cyclic dinucleotides) and a PD-1 blocking antibody. These nanomaterials effectively targeted the tumor microenvironment (TME), significantly enhanced T-cell infiltration and antitumor efficacy, thereby overcoming resistance to monotherapy with immune checkpoint inhibitors (ICIs) (1–3, 5) (**Figure 1**).

**Figure 1:**
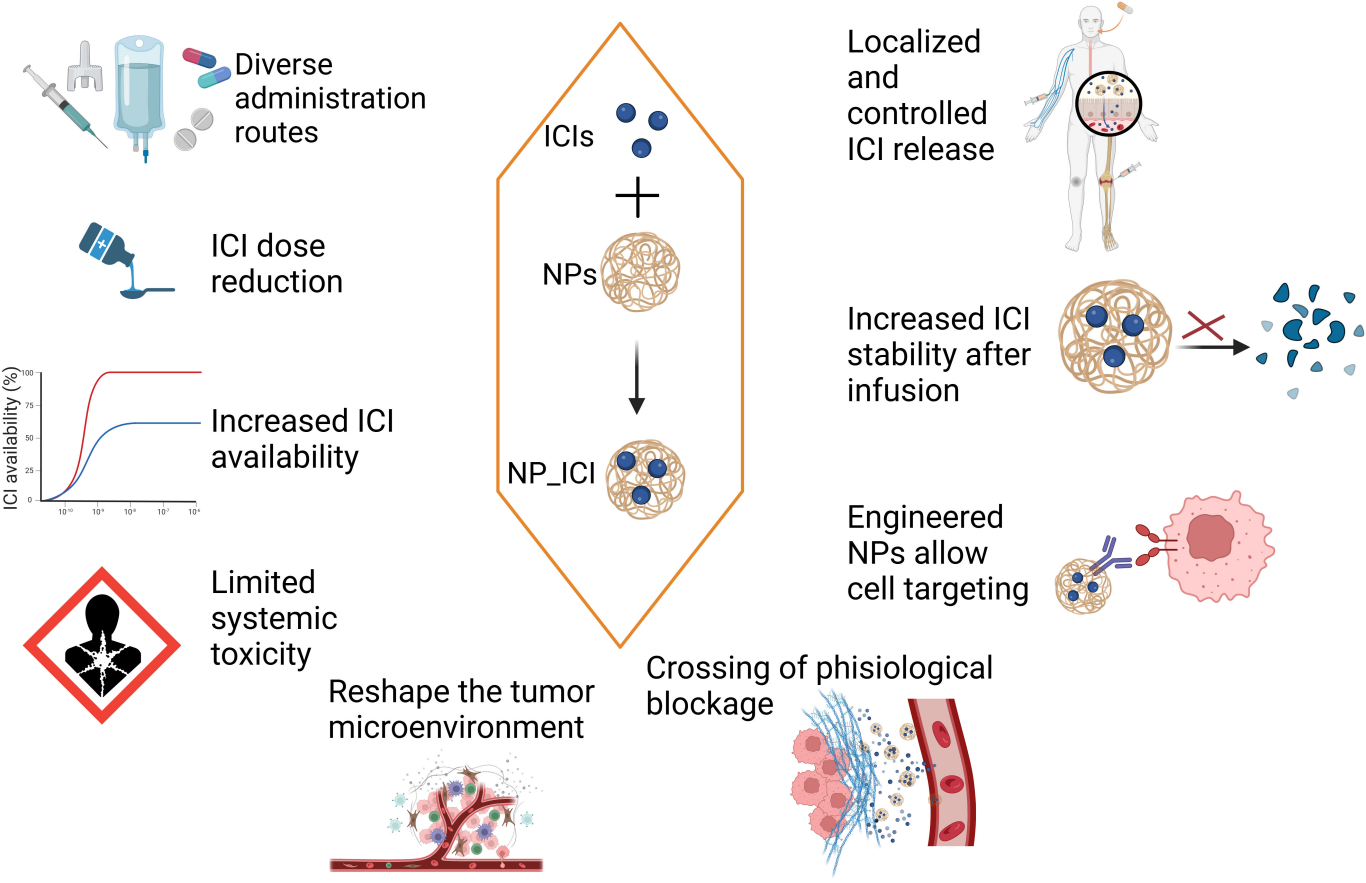
The potential of combining nanomaterials with ICIs in cancer treatment.

Secondly, nanomaterials can enhance the immune activation effects of ICIs. Immune suppressive factors in the tumor microenvironment (such as TGF-β, IL-10) may weaken the efficacy of ICIs. Nanomaterials can carry immune activators (such as TLR agonists or STING agonists) and, when used in combination with ICIs, can enhance their immune activation effects. For example, Meixi Hao and his/her team developed a novel neutrophil-based cell drug (NEs@STING-Mal-NP) that combines liposomal STING agonists with the surface of neutrophils. NEs@STING-Mal-NP effectively activate the STING pathway and reinvigorate the tumor environment by converting macrophages and neutrophils to antitumor phenotypes, promoting the maturation of dendritic cells, and enhancing the infiltration and tumoricidal ability of T cells (6).

Furthermore, nanomaterials can improve the persistence of ICIs. By combining carboxymethyl chitosan (CMCS) with bioadhesive nanoparticles based on polylactic acid-hyperbranched polyglycerol (BNPs), Shu Liang and his/her colleagues developed an injectable nanohydrogel (7). This study found that the BNP/CMCS nanohydrogel could maintain the activity of ICIs and continuously release the drug over 7 days.

Although the combined application of ICIs and nanomaterials shows great potential, there are still many challenges in clinical applications:

1. The physicochemical properties of nanomaterials, including but not limited to size, shape, and surface charge, have a significant impact on their binding mode and stability with ICIs. This, in turn, affects drug release and therapeutic efficacy. For instance, complexes formed by nanoparticles with extremely small sizes following conjugation with antibodies may lack stability. Given their relatively high surface energy, these nanoparticles are prone to aggregation or dissociation within the physiological environment. Such instability can lead to the detachment of antibodies from the nanoparticle surface (8). Moreover, nanoparticles of overly small sizes may be rapidly cleared from the body through renal filtration. This shortens their circulation time *in vivo*, thereby diminishing the efficiency of drug delivery (9).
2. The long-term safety issues of nanomaterials involve two aspects: the potential toxicity of nanomaterials themselves and the synergistic toxicity caused by their combined application. Some nanoparticles may accumulate in the body, leading to chronic inflammation and tissue damage. For example, after entering the body, synthetic amorphous silica nanoparticles (aSiNPs) can accumulate in cells through active endocytosis and passive diffusion, distribute in almost all organs, and cause inflammation and tissue damage directly or indirectly (10). In addition, ICIs themselves may cause immune-related adverse reactions, such as autoimmune diseases and endocrine disorders. The addition of nanomaterials may further aggravate these adverse reactions or trigger new toxic reactions. For example, nanomaterials may change the distribution and metabolism of ICIs in the body, resulting in an excessively high concentration of the drug in certain tissues and increasing the risk of adverse reactions. The interaction between nanomaterials and ICIs may also affect the balance of the immune system, leading to excessive immune activation or immunosuppression.
3. Nanomaterials may be recognized as foreign substances by the immune system *in vivo*, thereby triggering an immune response that results in their rapid clearance. This consequently reduces the effectiveness of nanomaterials as carriers for ICIs. Moreover, the immune response may also induce inflammatory reactions and tissue damage, which can compromise the patient’s health. For example, the amino groups on the surface of polyethyleneimine (PEI) nanoparticles endow them with a strong positive charge. Once introduced into the body, they readily and rapidly bind to serum proteins, leading to robust complement activation and an intense immune reaction. Lipid nanoparticles can adsorb plasma proteins in the bloodstream, forming a protein corona. This formation alters the surface charge and particle size of the nanomaterials, thus influencing their distribution and targeting ability *in vivo* (11).

Future development trends encompass intensifying the research and innovation in the development of nanomaterials and ICIs, as well as creating novel nanocarriers. In particular, the exploration and development of natural nanomaterials, such as cell membrane-derived vesicles (12) and peptide assemblies (13) are emphasized to enhance their therapeutic efficacy and safety. For instance, efforts will be made to design nanocarriers with higher targeting specificity and lower toxicity, and to develop new types of ICI drugs capable of overcoming tumor immune escape. Concurrently, investigations into novel mechanisms of action and therapeutic targets are essential, which can offer new perspectives and methodologies for the advancement of cancer immunotherapy.

## Data Availability

The original contributions presented in the study are included in the article/supplementary material. Further inquiries can be directed to the corresponding author.
